# Travelling to die: views, attitudes and end-of-life preferences of Israeli considering receiving aid-in-dying in Switzerland

**DOI:** 10.1186/s12910-022-00785-w

**Published:** 2022-04-30

**Authors:** Daniel Sperling

**Affiliations:** Department of Nursing, University of Haifa, Haifa, Israel

**Keywords:** Suicide tourism, Aid-in-dying, Death and dying, Israel, Qualitative research, Dignitas, Suicide

## Abstract

**Background:**

Following the increased presence of the Right-to-Die Movement, improved end-of-life options, and the political and legal status of aid-in-dying around the globe, suicide tourism has become a promising alternative for individuals who wish to end their lives. Yet, little is known about this from the perspective of those who engage in the phenomenon.

**Methods:**

This study applied the qualitative research approach, following the grounded theory tradition. It includes 11 in-depth semi-structured interviews with Israeli members of the Swiss non-profit Dignitas who contemplated traveling to Switzerland for aid-in-dying.

**Results:**

Seven themes emerged from the data analysis, including health and functioning; feelings regarding survivorship and existence; interacting with the health sector; attitudes regarding death and dying; suicide; choosing death; and choosing suicide tourism. A significant portion of the participants had experienced suicidal thoughts and had even previously attempted suicide, some more than once. Most of them referred to chronic illnesses, functional disability, and social isolation. They understand suffering within the subjective dimension, namely only by the person who is actually subjected to the disease, ailments, and disability. Participants regarded aid-in-dying in Switzerland as positive thanks to its guaranteed outcome: "beautiful death", compared to "disadvantaged dying" which places a burden on the participants' loved ones throughout the prolonged dying. Most of them do not necessarily want to have their loved ones beside them when they die, and they see no significant meaning in dying in a foreign country to which they have no emotional or civil attachment.

**Conclusion:**

The desirable approval or tragic refusal by Dignitas to participants' requests for suicide tourism enhances the paradox between the perception of aid-in-dying as a mechanism for fulfilling controlled death and its bureaucratic and materialistic characteristics specifically reflected in a paid, formalized approach to aid-in-dying that cultivate dependency and collaboration.

## Background

Aid-in-dying practices and legislation have expanded dramatically around the world over the last twenty years [1]. As additional countries legalize aid-in-dying (also known as assisted dying or assisted suicide) and consider extending the application of existing laws, this practice becomes an integral component of societies’ expectations, rules, motives, and symbols, representing a cultural desire to shorten the dying phase. While this may be a response to (or the antithesis of) medical efforts to prolong life, it impacts the benefits of a recognized and perhaps longer dying period [2].

Yet, aid-in-dying is still prohibited in many Western countries, and is criticized for three main reasons. First, as the prognosis of remaining lifetime and available treatments is uncertain, patients could receive support to help them stay alive and even improve their condition. Second, if aid-in-dying is permitted, then palliative care will be less prioritized and accessible to those who wish to die "naturally", with no self-intervention. Third, the practice of aid-in-dying violates the biomedical and bioethical principles of respect for life, thereby placing physicians in the unacceptable position of (indirectly) ending their patients’ lives, by prescribing lethal doses for example [3].

Following the increased presence of the Right-to-Die Movement, improved end-of-life options, and the political and legal status of aid-in-dying around the globe, suicide tourism has become a promising alternative for individuals who wish to end their lives. Yet for many, this must be performed outside their homeland, where receiving help for such actions (mainly through the prescription of lethal drugs) is illegal. Indeed, the evolving phenomenon of suicide tourism, which has drawn much criticism and debate [4, 5], has also attracted interest in their academic fields of medical ethics[6, 7], tourism [8–10] and cultural studies [11].

Most of the (relatively few) countries where aid-in-dying is legal only permit this practice for their own residents. For example, the USA Oregon Death with Dignity Act requires the patient to be a resident of Oregon; Canadian law only permits aid-in-dying for permanent residents of a given Province who contribute to and are insured by the Canadian healthcare system [12]. However, the law in Switzerland does not include such limitations. Moreover, Swiss law does not require an ongoing relationship between the patient and the prescribing physician, as is required in Luxemburg or the Netherlands. As such, many non-residents opt for aid-in-dying in Switzerland. The process is relatively easy and is usually facilitated by Swiss Non-profit Right-To-Die organizations, especially the renowned Dignitas [13].

In November 2018, the Swiss Academy of Medical Sciences [14] issued five requirements for aid-in-dying in Switzerland: [1] the patient has the capacity to issue such a request—a capacity that must be assessed and documented; [2] the patient's desire is independent and well-considered; [3] the symptoms of the patient’s disease or impairment cause the patient intolerable suffering; [4] the patient has sought other medical treatment options that were ineffective or unacceptable; and [5] the patient's request is understandable, based on the patient's history and repeated discussions with the physician who agrees that aid-in-dying is suitable in this case [14].

To truly understand people who no longer wish to live, one has to inquire not only about their motivations, but also about their personal histories, beliefs, and intentions underlying their request [15]. A systematic review of qualitative and survey data in 110 studies published by 2011 revealed a wide range of perspectives through which people (patients, carers or the general public) reflect on aid-in-dying. For example, poor quality of life, quality of death, and the ability to choose or alternatively potential abuse of aid-in-dying, and the importance of individual standpoint, especially moral or religious views, or previous experiences of death and suffering of loved ones [16].

A more recent systematic review covering qualitative studies from 2001–2016, on the expression of people's wish to die due to life-threatening conditions, found five major themes [15]: [1] Suffering was an overarching theme and a common denominator for understanding the other themes, and included physical suffering as well as psychological, social, or existential suffering; [2] Reasons for wishing to die, including physical, psychological, emotional, and social reasons; [3] Meanings attributed to the wish to die, such as a cry for help, a preferable alternative to suffering, sparing others having to care for them, a way of preserving self-determination, and an expression of another form to live; [4] Functions that are addressed through the wish to die, such as means for communicating feelings, thoughts, and wishes, and means for maintaining control over one's life; and [5] the feeling that time is running out, death is imminent, and we should be ready for it.

Attitudes expressed in previous studies among people seeking death include unbearable suffering, dependency, burden, and loss of self. The emotional, physical, psycho-social, and existential factors that were found were consistent regardless of social, economic, legal, or health-related contexts [16]. Gaignard and Hurst [17] found similar themes in interviews conducted with right-to-die professionals and volunteers regarding existential suffering in relation to aid-in-dying in Switzerland. Moreover, studies show that people who consider life and death decisions also consider the views of their social environment, specifically their loved ones and consult with them [18], even focusing on their loved ones’ needs more than on their own needs as the dying person [19].

Few studies have examined family involvement in aid-in-dying [20–22]. Fewer explored the views and attitudes of those considering aid-in-dying [15, 23, 24]. A recent study of observational netnography examined five open Facebook communities for early-stage dementia patients and their caregivers (formal and informal). The findings show that existential distress caused by loneliness, uncertainty about future decline, fear of losing meaning, control, and freedom of choice, and fear of a prolonged death constitute major reasons for people considering aid-in-dying [25]. Moreover, interviews with people requested aid-in-dying or euthanasia in the Netherlands reveal a range of fears relating to future suffering associated with the course of illness, physical symptoms, the dying process, loss of autonomy or self-determination and anxiety [23]. Based on evaluations of viewer's comments on YouTube videos relating to physician aid-in-dying, Yu et al. [26], suggest three themes for explaining the public intrinsic motivation for engaging in this practice (avoiding pain during the dying process, pursuing meaning in life, and maintaining human rights [i.e., the right-to-die]), and a theme for explaining extrinsic motivations (religious or social considerations). Extended analysis of these data also referred to humane acts (comparing suicide travel to the practice of animal euthanasia, thereby suggesting that if pet owners have a right to euthanize animals against their will, some of the same moral values should apply to human beings) and legal issues regarding the criminalization or legality of aid-in-dying [27].

Furthermore, analysis of 1,231 online reviews from the five most highly-viewed YouTube videos about suicide tourism revealed four dimensions of travel constraints: perceived incapability of being able to complete the necessary tasks for traveling, especially due to their illness or disability; lack of suitable travel agencies and services, limiting access to such services; lack of personal support; and complex travel decision- making stemming from the individualistic and collectivist nature of the decision, with a focus on the effect and implications of such a decision on others [28].

The only published study that this author found to explore the views and attitudes of those who considered traveling to Switzerland for aid-in-dying is based on interviews with seven British patients who considered such a plan, combined with participant-observations at a do-it-yourself self-deliverance workshop [29]. The findings show that the decision to travel to Switzerland for this purpose stems from a range of reasons, from a perception of a last resort to one that relates to the aesthetics of the good death, thereby attaching values to the physical and practical experience of such an event. The participants in the study noted that they were unable to make sense of their suffering (regardless of their degree of religious faith); as such making plans to travel to Switzerland and end their lives—a mode of death which entails some degree of control, certainty, and resolution—would thereby restore some such meaning.

In Israel, issues and practices pertaining to end-of-life care are regulated in the Dying Patient Act (2006) by professional guidelines issues by the Israeli Medical Association (2019) [30]. The law reflects the result of deliberations of a public committee and a compromise between religious, ethical and professional key players. It demonstrates the unique cultural and religious aspects of the Israeli society influencing its liberal values and practices, characterized by a mix of orthodoxy and secularism and of communal paternalism with individualism and permissiveness [31]. Moreover, it mirrors the primacy of the principle of sanctity of life especially in Orthodox Jewish ethics over other ethical principles which are mentioned in the law, specifically prevention of significant suffering, quality of life and patient autonomy [32]. The law holds that withholding life-sustaining treatment is allowed under specific circumstances. Advance directives are also legally valid but only with regard to dying patients, namely patients who are in their last six months of living [33]. Aid-in-dying is legally prohibited, although few bills to legalize it were initiated in recent years but did not lead to change of legislation [34]. Finally, the law acknowledges the right to receive palliative care and the duty to offer it. It holds that physicians must do whatever they can to ease the pain and suffering of a dying patient, even if it involves reasonable risk to the patient's life. Included in this duty, the obligation to also care for and promote the wellbeing of the patient's family.

Professional guidelines published by the Israeli Medical Association acknowledge that life and death are part of life and that physicians should be aware of their own values and the patient's values with regard to end-of-life treatment. The guidelines stipulate that life-saving treatment, including intubation should not be provided in all and every situation, and that physicians should consider providing care that improves the patient's quality of life when the alternative for that is futile treatment [30].

The literature lacks studies on the views and attitudes of people who seek aid-in-dying in general, and through traveling to another country in particular—including the meaning and significance that such a plan has on them. To close this gap, this study presents the following research question: What are the attitudes, perceptions, and challenges of Israeli members of Dignitas regarding their plans to travel to Switzerland to receive aid-in-dying. To answer this question, the research examines and presents the stories, needs, difficulties, desires, and accomplishments encountered by these individuals. Finally, this study maps their views of death and dying in a foreign country and analyzes the societal and State role in this phenomenon. In-depth discussion of the legal and practical implications of these narratives and meanings beyond the goals of the current study.

## Methods

The study applied the qualitative research approach, following the grounded theory tradition [35]. The data was collected through in-depth semi-structured interviews. The research population included 11 adult Israeli residents who were members of Dignitas. Using purposive sampling, the participants were recruited based on their ability to elucidate the research phenomenon [36]. The sample did not intend to provide a representative sample, but to offer various elements and positions that are relevant to the research question [37].

The author used the assistance of Dignitas in recruiting participants as the relevant research population usually prefers to remain anonymous, fearful of possible criminal investigation or social condemnation for themselves or their families. The researcher therefore composed a letter, inviting members of Dignitas to participate in the research. The organization emailed this letter to its Israeli members. On December 31, 2019, there were 138 Israeli members registered with Dignitas, yet the author does not know whether the letter was emailed to all Israeli members or only some. Moreover, the organization was only informed of the general research question. It was not party to the interviews, data collected, or the author’s analysis and interpretations. The members were encouraged to contact the researcher by phone or email for further information.

A total of 15 people contacted the researcher with questions and expressed an interest in the study. After an initial conversation, three of these members decided not to participate in the study, either due to lack of interest, being fearful of participation, or feeling that the topic would pose an emotional burden for them. Two people originally agreed to participate in the study but encountered scheduling difficulties and eventually did not get back to the researcher. Participants were recruited until thematic saturation has been achieved. In one other case, the interview took place with the husband and the wife–both members of Dignitas. As such, 11 Dignitas members participated in the research.

A five-section interview guide was developed especially for this research, based on relevant literature that is mentioned in the last section. The interview grid contained a few general questions, and then was constructed inductively, based on the interaction between the researcher and participants. Part I included biographical questions regarding the interviewees, such as age, education, and work experience. Part II referred to the health and functional status of the participants, with questions such as, "Please tell me about your disease" and "How well do you function on a daily basis?". Part III, which consisted of the largest number of questions, presented questions relating to participants' travel plans to Switzerland. For example, "How did you hear about Dignitas?"; "What are your expectations from this trip"; "What preparations have you made for this plan?"; and "Which challenges have you encountered while preparing for this?" Part IV focused on the participants' attitudes towards death and dying, as well as their personal encounters with this matter. For example, the participants were asked, "What does death mean for you?"; "Have you ever been near someone close who was dying? If so, what memories do you have from this experience?"; and "What expectations do you have from your own dying?". Finally, Part V of the interview guide enabled the participants to address issues that were not raised by the researcher during the interview or that they would like to ask the researcher in light of the interview.

The author of this study was the sole researcher and interviewer. The interviews were all conducted in Hebrew and lasted 72–157 min. Of the 11 interviews, 10 were recorded and transcribed. All interviews were conducted in the chosen place of participants, mostly in their homes. One interviewee refused to have the meeting recorded due to legal uncertainty and fear of social implications. In this case, the researcher made hand-written notes of the interview. For all interviewees, identifying data was disregarded wherever possible, to maintain anonymity.

Prior to the interviews, the participants were informed about the research process, objectives, expected benefits, and possible risks. Following this information, each participant either declined or consented to participate. When signing the informed consent form, the participants were able to add specific requests regarding their agreement, such as requesting to review their transcripts, be informed of the research findings, or be notified about the article publication. The research program was pre-approved by the Ethics Committee of the Faculty of Social Welfare and Health Sciences at the University of Haifa (#2018–41, dated 15 March 2018). The research was funded by the Interdisciplinary Center for the Study of Dignified End-of-Life Research at the Hebrew University of Jerusalem.

Data analysis was chosen based on the Ground Theory Approach. The study began with a sociological perspective of the phenomenon of suicide tourism, guided by an attitude of openness and inquiry to ensure the constant emergence of concepts. The generating of various categories consists of frequent comparisons between and within incidents and concepts, through a process of theoretical coding that establishes meaning, interpretation, and understanding of the researched phenomenon [38]. Analysisconducted in four stages: [1] All interviews were read in full, and initial coding and categorization was performed—resulting in 257 codes; [2] second coding and categorization resulted in 196 codes; [3] th code categorization within and between interviews was performed and category titles were amended and unified, while ensuring that the original understanding and meaning were not altered. Review of the categories led to the omission of a number of categories that seemed to be out of the ordinary and had no significant contribution to the research question. Interpretation of the data was based on a phenomenological-interpretive approach with a focus on the participants' claims, explanatory logic, biographical contexts, and cultural frameworks of knowledge; [4] Major themes identified from the interviews were developed and re-organized into a scheme of codes and central themes [39, 40]. Findings were arranged in final categories and themes, with a hierarchy being determined and justified, and core categories being identified. Throughout the whole process of data analysis, the author ensured reflexivity and discussed some of the major findings anonymously with two colleagues who are expert in qualitative research. The study and data reporting were conducted in accordance with the Consolidated Criteria for Reporting Qualitative Research (COREQ) [41].

## Results

The 11 participants varied in gender, age, marital status, level of educational, occupation, health status, and place of residence. They included nine women and two men, aged 35–81. All of the participants were Jewish, most of them were secular Jews, and two interviewees regarded themselves as traditional. Three were married, two divorced, one widowed, and five were “legally” single (two of whom were in relationship). Their education ranged from high-school education to a PhD and they lived in a range of towns and cities across Israel. The data analysis resulted in seven major themes: *health and functioning status*; *feelings pertaining to survivorship and existence*; *experience with the health sector*; *attitudes regarding death and dying*; *Suicide*; *choosing death*; and *choosing suicide tourism*.

### Theme 1: health and functioning status

This theme includes two major categories: *health status* and *functioning status*. All participants referred to their declining *health status* through associations such as feeling weaker, being less autonomous, being physically impaired, or not being able to walk or speak. They all mentioned one major health condition from which they suffer, such as cancer, Myelodysplastic syndromes (MDS), or heart failure. Most also referred to additional ailments that added to their condition. Some elaborated on their hospitalization experiences and medical treatment they received (physical, mental, or both). Few of them mentioned the side effects of the main disease or its treatment, such as having dry eyes due to MDS or suffering from dizziness and nausea following chemotherapy. Two of the participants were also suffering from depression. Another participant had endured extensive mental-health hospitalization and treatment.

However, despite their harsh medical conditions, the participants tended to speak more about their inability to *function*. Most emphasized their being highly independent people who lead independent lives. They shared their desire to maintain their freedom of choice, to be able to decide for themselves about their own lives until the day they die. As a result of their deteriorating health, they talked of their decreasing independent functioning. Some provided concrete examples of tasks that they are no longer able to perform without the help of others, such as walking steadily, bathing, reading, speaking, driving, shopping, gardening, and even picking up an item from the floor.

In her interview, D., a 35-year-old woman with a number of medical conditions and complications, expressed what it means for her to lose her independence and having to be dependent on her close friend, Z.:

I wouldn’t wish my situation on anybody, this continuous situation of dependency. I can't leave my city. I even can't leave my neighborhood. I’m dependent on an organization to bring me food. I’m dependent on my cleaner, who has become much more than just a cleaner for me. He hardly cleans for me. He cares for me. He fetches things for me… takes things down for me, fixes things. He's everything to me. He does my grocery shopping. I am dependent on Z. who himself is deteriorating due to my numerous requests. He himself is an old man and cannot be beside me every day, because let's be honest… Is this living? And then what? And then what? Tell me. What will happen if something happens to him… You won’t find [someone like him] anywhere… I’m fed up being dependent on others. I have a volunteer who buys my medicines at the pharmacy. She comes once a week. I am dependent on volunteers… Why should I want this life? Why do I need to continue living this life? Just because you think I should continue living? No. It doesn’t suit me.

Most participants expressed an aversion from receiving nursing care, being bathed or changed by others. One interviewee, who lives in a luxurious nursing home, expressed how living in a nursing home actually imposes suffering:

There is, in some respect, quality of life here, no doubt about it, if you don't look to the sides… Living here means saying goodbye to everything, to everyone: family, kids, being close to somebody. It's a shock… I’ve already been living here for nine years. This is a home that offers quality of life. Very nice. But I see the decline in all the residents in this building. It can be as fancy as you like, but time does its own thing. People who were 80 [when I first arrived] are now almost 90. They all need wheelchairs or walkers. It's not fun seeing the physical and cognitive decline of these people, despite the [so-called] quality of life.

### Theme 2: feelings pertaining to survivorship and existence

Research participants who referred to this theme expressed their feelings through 11 different categories: *general feelings*; *difficulties*; *unsatisfactory life*; *hopelessness*; *life without essence*; *despair*; *giving up on life*; *quality of life*; *ageing*; and *pain and suffering.* In *general*, the research participants did not convey feeling sad about their situation. Few of them expressed a general *difficulty* with the outside world, manifested in distrust in other people and being disappointed with them. They referred to situations of injustice, lack of legal enforcement, and the covering up of mistakes made by medical professionals or the police, that accords with the maxim "You will protect me, and I will protect you". Not because they "have something against nature, plants, or animals, but because they have something against people; people are arrogant and patronizing towards others who are suffering, who are weak or different." Moreover, some specifically conveyed their life being *unsatisfactory* as well as feelings of *hopelessness*. In the words of D.:

Twelve diseases and six or seven neurological and orthopedic problem. Is that not enough? Is fibromyalgia, which is like 10 diseases, not enough? I should have killed myself many years ago… I regret these twenty years where I hardly resisted [killing myself]. I told myself that it might be OK… that this thing, which I didn't even know what to call it, would pass. This disease. I did know its name. It might pass. Then I told myself that if I receive a diagnosis, then I will be ok. Then I told myself, OK, the treatments are helping, I will go on with this. But then I saw that this really doesn't work. Then I told myself, there were many things, all the time there were many false hopes, things that I didn't even know were false. But it is human nature to try to hold on to hope. I don't choose hope. I don’t want to. It's my right. I think that after twenty years, I’ve earned it.

Suffering from advanced bone cancer, 77-year-old I. referred to her feelings of living life without essence: "I’m like an ostrich. I hide my head in the sand until the sun comes out, and then it’s lunchtime, and then I go to sleep at 21:30"; Another participant, G., a 81-year old woman who suffered from heart failure and was severely paralyzed, expressed a strong feeling of despair, admitting that her situation is so bad, empty, and unnecessary with nothing to look forward to. A third participant, A., a 35-year-old woman with a history of extensive mental-health hospitalization and treatment acknowledged that she had given up fighting to live, *given up on life itself*, having regarded on her situation as pointless, with a physically presence but lacking all worth.

Many participants referred to a decline in their quality of life. C., for example, a 66-year-old woman suffering from post-traumatic neuralgia, described how her ability to enjoy life has been compromised:

I can't sleep, I can't lie on my side, I can't go out into the sun, I always need an umbrella outside. The moment I’m exposed to the sun, I experience sharp pain. I live on medications, even medical cannabis. I try to come off one kind and use another because it's not healthy. Look, you can’t go out into the sun. It’s awful and it interferes with many things. I’m sitting talking to you and all of this area is… painful. I can't talk much because the muscles are working…

The findings of this study indicate that declining *quality of life* was associated with declining mobility, being imprisoned in a body, and not being able to care for oneself. Some participants claimed that while this may be inevitable with ageing, they do not wish to experience this due to illness. Moreover, declining quality of life was also connected to the concept of *ageing* with dignity. One participant stated that nowadays, people hardly age with dignity at home. At a certain age, they end up living with another person who helps them, while beforehand, they conducted full independent lives. This participant was also critical of such situations:

Something is wrong with this. I think people have to know, have to decide when they want to go. This can be at the age of 70 and it can be at the age of 90. The question is whether they are even given this option.

Some participants associated quality of life with their overall feelings about life with *suffering*. One participant, B., a Holocaust survivor with advanced spine proteus and digestive system dysfunction, admitted that her pain is so bad she cannot even scream. She simply jumps in pain and waits for it to pass. Indeed, most participants in this study were on strong painkillers, including morpheme and medical cannabis.

F., a 48-year-old woman with stage four colon adenocarcinoma mentioned that she had liver metastasis and that dying from liver dysfunction is excruciatingly painful and she want to die before she gets to that stage. She is already taking the maximum dosage of painkillers and she knew that there would come a time where they no longer help her. A similar idea was expressed by E., a 79-year-old woman who said, "I don't want to suffer. I wouldn't have wanted to suffer, and I would have been ready to leave this world elegantly without burdening anyone, without causing anyone suffering." F. also referred to one of Dignitas’ statements, whereby the choice is not between life and death but between the type of death:

There is no choice between living and not living as we are all going to die. The question is how. If could choose life, I would choose life… A person should live life in comfort. In no way is that related to suffering – mental or physical.

### Theme 3: experience with the health sector

This theme includes six categories: *hospital experience*; *inefficient mental care*; *communication with healthcare providers*; *disappointment with healthcare providers*; *inability to deal with doctors*; and *trust in medicine and healthcare providers*. Although a number of participants referred to their *hospital experience*, F.’s description was especially interesting with regards to suicide tourism, as it also relates to a category that will be discussed below: dying a private / non-embarrassing death:

One of my friends was in hospital on his birthday. His relatives came to visit him… with balloons and kids, and the patient sharing his room dies at that exact moment. My friend’s relatives started screaming and crying. This didn’t make sense to me… I don’t want die next to strangers, who know nothing about me and don't care about me… When I die, I want privacy, or at least to be among people I love, people who I would want to see, people that I trust. I don't want strangers with their own problems near me.

In some of the interviews, participants expressed disappointment in the medical staff they encountered or the treatment they received. Few talked about *inefficient mental care*. In one interview, A., a 35-old-woman suffering from depression, schizophrenia, and other mental conditions, attested to the mistreatment of patients that she witnessed during her hospitalization, and how the staff are mechanical, cold, and disinterested when interacting with the patients. Others referred to *miscommunication* with healthcare providers or overall *disappointment* with them. They also experienced being transferred from one provider to another, trying ineffective medicine, and encountering a lack of any connection or empathy. With tears in her eyes, one interviewee voiced her inability to *deal with doctors* and hospitals and saw no real purpose in doing so. On the other hand, some participants did convey respect *and trust in medicine and in healthcare providers*; they were not critical of the care they had received and, instead accepted their situation seeking to more fully control it.

### Theme 4: attitudes on death and dying

Twelve categories are included in this large theme: *the right to die*; *the legal status in Israel*; *the freedom to decide*; *end-of-life planning*; *fear of death*; *the meaning of death*; *early death*; *dealing with and talking about death*; *religious influence*; *burial*; *hospice*; and *end-of-life fantasy*.

Some participants articulated their situation through the concept of *their right to die*, equating their right to die with the notion whereby people should not have to cling on to hope, but deserve to be able to choose death. In addition, many addressed the legal prohibition of aid-in-dying in Israel. They criticized this *legislation*, claiming that this poses a burden for them, as they are required to choose an option (i.e., traveling to Switzerland) which otherwise would not have been their first choice. Reference to the legal status was also made with regards to medical practice. Some participants conveyed that they are unsure whether some medical doctors in Israel do assist their patients in dying or easing their pain with medication until they die—despite the first being illegal. Some even claimed that the legal position regarding aid-in-dying (and euthanasia) is influenced by religion and has not changed for years. However, most accepted this position and did not argue against it.

According to these interviews, the right to *freely decide* about end-of-life issues derives as life being their own and people should be permitted to make decisions about their death just as they are allowed to make choices regarding their lives. However, despite such choices being valuable and private, most people do not think about them when they are young and/or healthy. When *end-of-life planning* is mentioned by participants, it is usually done within concrete contexts, such as advance directives and the Israel Society to Live and Die with Dignity [ISLDD]. Some of the participants voiced concerns that not all doctors respect advance directives. One interviewee even said that she cannot really count on that, "because you may be paired up with a doctor who sees this as a betrayal of the Hippocratic oath." They expressed fears that in cases of a stroke, for example, they will automatically be transferred to a rehabilitation center where they will be retaught how to walk and speak. As articulated by C.:

If, for example, I am hit by a car in an accident. I’m lying on the road, and I can't speak. The ambulance comes along and they immediately give me whatever they’re supposed to, right? The fact that in my bag is a Ministry of Health card [for no life-sustaining measures] and another card… The Ministry of Health made good progress, the ISLDD pushed them. I’m very proud of them for that. I don't know how many paramedics would even look in my bag for these cards. That’s exactly what I’m concerned about.

Research participants referred to common perceptions about death, such as people can "faint when hearing about death." People *fear death* yet are intrigued when others discuss it: "They may think that you have a personal issue. What, are you sick? Is that why you’re discussing death?." Most participants stated that they were not afraid of dying. For J., a 73-year-old man suffering from MDS, death has no *meaning*. If anything, it has meaning for those who are close to him. Others, however, referred to various meaning of death: Death is "a final matter, equivalent to not being here before life." According to one interviewee, a physician:

Death is the end. That’s it… You stand near the mortuary table and you perform the autopsy. And you see everything, even the blood you cannot see. You see flesh and all of the human anatomy. But the human being who was here, he did what he did and now he’s gone. It's difficult to say this, but it's very realistic. We always searched, during these autopsies, for the soul. We never found it. There are so many things that we cannot explain, but death is something final.

Another interviewee said that death is a "passage to permanent accommodation," while a third participant regarded death as "freedom, an exist point from this life… pleasure, release. Certainty. [There’s} no chance that I’ll still be handicapped, no chance that someone will see me and save me. A certain death." E. also expressed a similar idea, referring to cases where people unsuccessfully attempted to kill themselves or be assisted by a medical doctor: death is "something that works out."

A different perspective linked death with privacy: Death is "being surrounded by people I love, people I want to see, people I count on. I don't want strangers with their own problems anywhere near me." For J., death also had a temporal dimension, as it can be an *"early" death*, namely before one has had time to do good things.

In this research, the participants admitted that they differ in their *dealing with and talking about death* (one participant even regarded herself as "abnormal" in this perspective). Feelings about death were also associated with and shaped by the participants' biographies. For example, one participant spoke of her position as a military doctor during the Yom Kippur War (1973). "There were dozens of cases where we walked through the streets and children yelled at us “angels of death”… I had to inform… families that their child or son or father or brother had died at war." She also referred to her experience as an internist at a hospital, encountering death everywhere.

Most research participants criticized the *religious* influence on attitudes towards death and dying. As C. expressed, "Judaism and Christianity took us off the normal path. Only the big religions sanctify life in this way." I. also held the religious Jewish society in Israel responsible for the current situation: "They will always find some justification in the religious scriptures for opposing suicide. I’m sure it's easy for them to explain why not, but it's not easy for them to address person's issue." That’s why I. refused to discuss her plans with the religious doctor who was treating her at the moment. E., who was raised in a secular family in a communist country said that all religions forbid the taking of one's life, including Christianity, Judaism or Islam. As such, she was afraid that her choice to travel to Switzerland for aid-in-dying might harm her family, especially her ultra-religious grandchildren with whom she has a good relationship.

Participants' attitudes on death and dying were also reflected in their views on specific issues, such as *burial and hospice*. One participant referred to burial as a waste of land. She claimed that burial is important to many people, as it guarantees a place to visit the dead after they die; she even confessed that she has been criticized for not having headstones at her parents’ graves. For another participant, burial is totally something for his wife to decide once his plan is secured. As he doesn't believe in after-life, no meaning is attached by him to burial. Another participant referred to home hospice, explaining that such care improves quality of life, alleviates pain, and eases symptoms. She said that if her situation were to worsen, then she would resort to this.

One participant expressed her *end-of-life fantasy*:

Every person should have the option of deciding when he is stuck and wants to go, to die… of buying a drug in the pharmacy or something like that, regardless of his medical or other conditions. Because I think that today, there are so few things that we can control… we’re responsible for our family, and for our work, and for our grandchildren, etc. …and here this is *something* that is really only mine. It's supposed to be only mine: and that [something] is life.

### Theme 5: suicide

This theme contains seven categories: *suicidal thoughts*; *suicidal attempts*; *healthcare providers' response to suicidality*; *the decision to not commit suicide*; *fear of suicide*; and *suicide of a healthy person*. Half the participants had had suicidal thoughts in the past, and a third had unsuccessfully attempt to kill themselves—either by hanging, jumping onto oncoming traffic, or taking a huge dose of sleeping pills. Some had attempted suicide a number of times. Those who spoke of suicidal thoughts had already experienced them early in their adolescence, following difficulties at home (such as fighting and violence within the nuclear family), unrequited love, a condition that led to shame and social exclusion (such as obesity), etc. During her interview, D. spoke of why she had jumped out onto the road when she was just 15 years old:

Health issues, I didn't understand what was going on, I mean what did I have? Why me? All the symptoms that started then. Why was I tired? Why couldn’t I study? Why did it take me so long to learn something? Why did I find it so difficult to walk from here to there? From one bench to another, I had to sit down and rest. Why was I finding it so difficult to go up [this hill]? I could just six months ago… Suddenly, all these disabilities that I didn't understand where they had come from. Everything. Everything together.

Some of those who had suicidal thoughts had expressed them to others. A., for example, told of how immediately after writing on an Internet forum that she was considering suicide, she was visited by the police. Another participant, who had attempted suicide a number of times and had been hospitalized in a psychiatric hospital, spoke of her healthcare providers' response to her suicidality. She said that the moment a doctor sees a suicide attempt in her medical record, everything changes for the worse. As a result, she felt that she had lost trust in them.

Participants also referred to changes in their suicidal thoughts or decisions over time. For example, D. described how she had been affected by her close friend Z., who had begged her not to commit suicide. Seeing his pain and knowing that he had already lost his brother and his parents, she realized that she could not image herself leaving him that way. In her interview, she openly talked about her desire to end her life:

All that fear of suicide. It's not a problem to decide to commit suicide. The problem is that it's not that easy to actually do it. People think it's easy, but it's not. You might find yourself with even greater disabilities. You might find yourself in an even tougher situation if your attempts to kill yourself do not succeed. And then what would I do? If today I’m at the mercy of so many people, what will I do if my situation worsens? I have a box full of medicine. But after poisoning myself in 2010, I realize that it's not that easy to kill myself. And it’s not like you can ask the doctor for a specific drug that will kill you, and quickly.

In his interview, K. explained his motivation to travel to Switzerland with his wife who had advanced spine proteus and digestive system dysfunction to end his life as well. An 81 year-old relatively healthy and functioning man, K. talked of the Swiss solution regarding suicide of a healthy person. Trying to imagine his life without his wife, he sees no sense in going on without her: "Everything we do together is meaningful, because we do them together." He adds that the decision to end his life stems not from a commitment to die together, but from the selfish desire not to spoil all the good that they had together by living an unsatisfactory life without her.

### Theme 6: choosing death

The following five categories are included in this theme: *a strong wish to die*; *feeling confident in choosing death*; *the choice of death*; *being close to death*; and *choosing death as a personal decision*. About half the participants expressed a *strong desire to die*, having exhausted all other alternatives. Some used harsh words to describe their lives at present, such as "I’ve lived 20 years of hell." Their strong wish to die is reflected in the concept whereby if someone wants to die, then they will die. As F. explained: "It’s a person's own decision about what to do with their life. Others can or can't help, can agree or disagree, but the final decision is his or hers."

The strong wish to die is also reflected in the desire to die *instantly*, as indicated by G.:

The thing I would like most is to dies instantaneously, now! I wish I knew doctors or people who would come, who were willing to come to my place and give me... This is what I want. To die in a moment… no doubt about it. Not even a single doubt. Because I live with this feeling that I have done whatever I could while I was alive and I have missed nothing. I won't miss anything if I do this.

The participants' strong wish to die is accompanied by *feeling confident* about such a wish. Participants proved to be very "rational", referring to medicine and statistics and showing disbelief in miracles. In general, the *choice of death* was understood as the person's elementary right that derives from their being alive. As conveyed by D.:

Everyone is entitled to choose their own death. It has nothing to do with being sick or not. That’s my belief. It is my belief that everyone is entitled to this. No one has the right to tell another person that they have to continue fighting. If a person wants this, is competent… let him die. What do you want? What? Why are you fighting?

Interestingly, the *choice of death* goes together with participants' being close to death-related circumstances or having frequently encountered them throughout their lives: through their own suicidal thoughts or attempts, the death of a loved one, advising others on death-related issues, or facing death during one's career. In general, the participants felt "friendly" towards death rather than feeling threatened or fearful of it.

Finally, according to most participants, *choosing death reflects a personal decision*, perhaps even the most personal one. As articulated by C., "people have to have the right to end their lives. This is so elementary, in my opinion. What else there is that it is truly only *mine*? That nobody else can claim from me? Just my life." A strong message was also conveyed by D.:

Nobody can determine who deserves to die and who does not. Only man himself can [determine this]. If the person has made this decision, then you can be sure that it was difficult for him to make this decision earlier. After battling for twenty years, believe me. I think I deserve this… Look, it goes against nature, everyone knows that, it goes against nature for a person decides to kill himself. I don't have to tell you this. Every researcher will tell you this, right?... Even if I see a snake… even if I want to die, I’ll be scared and run away, right? Because it's automatic, it's not a matter of thought. Right? But if a person decides to give up… then that person has had enough. I feel that I have had enough.

### Theme 7: choosing suicide tourism

This theme consists of the most amount of information and findings obtained from the interviews, grouped into the following four major categories: *decision-making pertaining to suicide tourism*; *personal thoughts concerning suicide tourism*; *preparations for this plan*; and *sharing information about this plan with others*. Interviewees first heard about Dignitas or the "Swiss option" through the media, experience of a close friend, or ISLDD. F. made the *decision to opt for aid-in-dying* almost as soon as she was diagnosed with cancer; G. made this decision when she returned from hospital and was forced to stay in bed as a result of her medical condition—an experience that she referred to as "a pressure cooker"; H., a 68-year-old single mother had made the decision fifteen years prior to the interview. For G., The "Swiss option" serves as "security so that one day I can say, enough, I can’t, I can’t, I’m in pain, I don't have the strength and I can’t exist with dignity… let's go there."

The research participants provided various explanations to support their decision. B., for example, said that she only wants to live "as long as living is worthwhile, pleasant, pleasurable. The moment our suffering exceeds the possibilities of things that we like to do—which are already slowly decreasing, then what's the point in continuing? For what? " C. regarded her decision as a gradual one that derived from her ageing and illness, symbolically saying that "things are moving in the wrong direction." For D., this decision was also justified by what she may expect in her future, the awful things that she anticipates. Both G. and J. emphasized their immobility and lack of independent functioning, as reasons for such a plan. H. referred to her helplessness and lack of dignity, mentioning that Dignitas could help her when there is no other way out; she didn't want her only son to "be involved in the difficult task of having to pull the plug on mom."

Despite their strong wish to die and their firm decision-making regarding the Swiss or aid-in-dying plan, few participants referred to certain factors that could have changed their decision. D. mentioned that if she had a larger and newer place to live, as well as sufficient income, there is a small chance that she would have given up this idea. E. mentioned her ultra-religious grandchildren and said that although she has not altered her plans, she does think that she needs to be considerate of their emotions, and as such, may possibly rethink her decision. Some participants mentioned *family* as an inhibiting factor. For example, C. who is childless said, "I can understand that for other people, children are a reason. People continue to live because they have children… People stay married because they have children…” One interviewee confessed that he has not yet told his wife about his plans to travel to Switzerland, assuming that she will never agree to this. Attempting to explain himself his wife's approach, he explains:

Part of it is mental, people don't want to stay alone, especially at this age. Part of it is also practical, as she is dependent on me in many aspects of life. Now, for lack of any other option, she is making an effort regarding practical issues, but it’s not enough. Her health is not that good. And all those practical things, you know, bank accounts, etc. We all have plenty of things. She doesn't know how to deal with them.

Contrary to these findings, two interviewees said that although they have a good relationship with their children and grandchildren, it is insufficient in compensating for their pain and suffering. One interviewee even admitted that when she is not around any longer, things will be easier for their children.

When asked about personal characteristics that enabled them to make this decision, interviewees provided various perspectives. For some, it was being realistic, knowing what ageing is associated with and preparing for it. One participant mentioned that she tends to focus on her own interests and feelings caring less about the opinions of others. Another mentioned being independent, acting according to their own desires.

Finally, participants were asked whether they had considered any alternatives to suicide tourism. Most said that they had examined such alternatives, but wished to refrain from a painful or very unpleasant act, such as hanging themselves, jumping from a high building, drinking cleaning products, or jumping in front of an oncoming train. With regards to the latter option, participants also explained that they would not want to harm others through their end-of-life act, such as the train passengers or driver. One interviewee had thought about the option of killing himself while sitting in his car and exposing himself to exhaust gas. While such an alternative can be executed independently, without the help of others, it is not easy to find a location where no one will suddenly intervene and put a stop to this act. The same interviewee also talked of ordering special sleeping pills via the Internet and using them in the future. This research participant had already contacted someone in the United States through an Internet search on "how to buy Nembutal pills". However, that person was unwilling to receive payment for the product through an Israeli credit card or bank transfer.

Another participant said that if she could have obtained a firearm license, she would have done so. She also said that if she is not approved by Dignitas, she will commit suicide in the Aokigahara forest or by an active volcano in Japan. A third participant mentioned she had thought of contacting a professional assassin and paying him to shoot her, but she admitted that she how no idea how to even start looking for someone like that. A fourth participant said that she had asked her sister and niece to go on a cruise with her so they can throw her out to the sea. However, this option was not practical because there are security cameras everywhere.

During the interviews, participants shared their *personal thoughts* concerning suicide tourism. One participant said that she wonders whether it will be an unpleasant, painful experience. Another participant was focused on which suitcase she would take with her to Switzerland so as not to arouse suspicion that she will not be coming back. She was afraid that someone will suspect what she is planning and will stop her. A third participant wondered whether Dignitas could provide a neutral person to accompany him rather than his family members. Few participants were occupied with the question of what will happen after they die. Those who raised the issue of burial showed no specific interest in being buried in their homeland. Two participants expressed their wish to donate their bodies to science, for the benefit of anatomy students, even Swiss students.

Many participants expressed fears regarding their plan to travel to Switzerland for aid-in-dying. Some were worried that in the end, they would not be able to realize their plan, especially due to their deteriorating health or ability to function that would prevent them from traveling. Others did not convey any fears at all. In general, the participants were fearful that their request would be rejected by Dignitas, that people will suspect what they are planning and will intervene, or that they will be unable to fly because of their health status. One participant was even afraid of the cold Swiss weather.

Some participants (not many) referred to fears associated with their engagement in an illegal or criminal act. One participant told of how she had contacted a family member who is a lawyer to enquire about the available options for someone in her position, knowingly that calling an outside lawyer could put her at risk. Another participant shared how following a conversation with her general practitioner (GP) about what she thinks of Dignitas, she was approached by the police and asked if she is alright and who she is living with. A third participant was afraid that if her companion accompanies her to Switzerland, he might be arrested upon his return to Israel.

The study reveals a wide spectrum of perceptions pertaining to the Swiss plan and its meaning. H., for example, explained how being a member of Dignitas and pursuing this plan provides her with a feeling of security, so that if one day she feels that she cannot go on, she will be helped. The plan to go to Switzerland was especially important for H., who is a 68-year-old single mother, because she could not imagine her son "pulling out the plug". For A. and C., the meaning of going to Switzerland stemmed from their not wanting to put others at risk or treating them unfairly if they were to retrospectively find out that their help had contributed to such an act.

The findings show that participants are particularly concerned with the notion of them dying alone versus dying beside someone close. F., for example, believes that a person's final moments should be private, so that the person can "fully concentrate" on the dying process. Other than one couple who wanted to die together—even though only one of them was terminally ill at the time, the participants emphasized that they did not necessarily want to die beside someone close, such as a child or parent.

Similarly, participants did not show any special desire to die in their homeland. For some, dying in a foreign country was meaningless. When asked how she felt about dying elsewhere, not in the familiarity of the city where she lives, surrounded by people and memories, H. replied:

I don't think of it that way because it's a fact… There’s no way of doing it here [in Israel]. There’s no way of doing it in Germany [where she was born and was a citizen], so… If I want to do this, I need to go to this place even if I have to travel there. It doesn't have anything to do with *home*.

For few participants, raising public awareness was also important. One participant explained that she plans on traveling to Switzerland with her photographer friend. The "deal" was that the friend would document the entire process with her camera and will then hold a photograph exhibition on euthanasia. When I asked this participant if she did not mind being photographed in this context, she replied that such an exhibition could help her friend as it would attract many people. She also hoped that when people saw the pictures, they would think more of people’s right to choose how to live or end their life, perhaps not having to seek remote places to do so. The desire for the process to be document was also seen in A.’s interview:

It’s important for me to find an author who will help me tell the story of my life, and the lives of many others… In this book, I will be able to openly discuss and develop things that I don't usually say. My story, which is the story of many other people… could fix many social things. I believe there are many people who are suffering, yet are unable to talk about the things that I have told you.

The interviews also explored how participants anticipate death through their plans to travel to Switzerland. A. said that this would be the greatest gift of all, something she has been occupied with for the past three years, alone, while dealing with extremely difficult issues. C. regarded this a form of "anonymous death" as something beautiful and pleasant. Similarly, F. imagined it as an "aesthetic death" linked to "traveling to Switzerland, enjoying the country for a few days, eating everything without worrying about calories, and then just drinking something with phenobarbital in and falling asleep.” In addition, this would be a death without suffering, a non-violent death. For both E. and H., death in this context would be dignified and peaceful, something that is of value to all human beings. In summary, the participants’ hope to die without pain or suffering, with ease, while enjoying whatever is left of their life until then.

As *preparations for executing their plan*, the participants had initially contacted Dignitas after searching the Internet for relevant information, discussing the subject with friends, prior to an important medical procedure, or directly after undergoing emergency surgery and realizing that life will never again be the same. Some referred to necessary circumstances before executing this plan, including being medically qualified, lacking healthy physical functioning but maintaining good cognitive functioning, being terminally ill and fully dependent on others; having the physical ability to travel to Switzerland; and being able to hold the glass and drink from it.

Most preparations for executing the plan can be divided into four major activities: [1] involving the family in the planning, mainly through informing close family members; [2] reading relevant information on the Internet and media or books that describe techniques for killing oneself (e.g., *Final Exit*, [42]); [3] completing other projects before carrying out the plan, such as traveling to exotic places; and [4] saying goodbye to loved ones (hoping that they will not dissuade the participant from carrying out their plan.)

The interviews conveyed how the participants need help and support to realize their plan. For example, translating documents for the application, completing the registration forms, making travel arrangements, receiving financial support for this plan, and even convincing their partners to support their plan. Moreover, as some participants lacked sufficient information or support, they sought people who had had experience with the Swiss plan or concept. One participant had encouraged two friends to also consider the Swiss plan for themselves. Another participant was willing to offer her support to other people who are contemplating travelling to Switzerland for this purpose yet lack someone to assist and accompany them throughout the process.

However, the participants described having encountered great difficulty in finding such support. For example, A. described how she begged for help, especially from professionals, especially lawyers and medical doctors, but was always rejected. The general response that she received was, “you can kill yourself if you want to, but no one will help you do so." Weeping, she told me that the only thing that had kept her going was her cat, who had now disappeared.

The issue of accompanying the participants to Switzerland was greatly addressed during the interview. Importantly, not all participants expressed a desire to be accompanied to Switzerland. One participant even said that “if a person wants to carry out this plan, she needs to get the job done alone.” Another participant told me that on the one hand, it would be nice to have someone accompany her to Switzerland and then inform others *post-factum*. Yet on the other hand, she was concerned that having someone accompany her would be difficult for her, as she would feel sorry for that person and might also put them at criminal risk. Another participant shared that she had discussed this issue with her close friend but had then been told that it would be very difficult for her friend to accompany the patient to Switzerland. One participant told me she wanted her son to accompany her and was confident that he would agree. She regarded this not as a form of help or support, but as a means for saying goodbye. Another participant said that he was looking for someone who would agree to accompany him, even for money, in case he regresses physically and is unable to travel alone.

Being in contact with Dignitas was described as being a long and complex process, with the organization requesting numerous documents that all need to be translated into English. The organization is perceived as demanding very precise requirements, but also very rigid. One interviewee explained that all communications are conducted through postal mail, or with email replies being anonymous due to the legal risk entailed in this practice. She also said that you cannot simply speak to someone there to explain your situation; most contact is relating to bureaucratic aspects such as required forms or documents. One participant found the slow speed of the organization to be difficult, and felt that she was not understood. Moreover, she was unsure to what degree she should inform the organization of her financial difficulties. Although she suffered from excruciating and debilitating pain, she did not have a terminal illness, and as such was concerned that her condition would not meet the organization’s criteria for approving her request. Another participant said that she had encountered language gaps, lack of communication, and lack of knowledge when contacting the organization. She was also worried about requesting a letter in English from her physician, which could disclose the real purpose of the letter and in turn put both herself and her GP at risk of taking part in criminal activity. On the other hand, one participant said that the organization’s rigorous selection process is actually advantageous, since its requires applicants to think and rethink their plan, to be certain that they wish to die.

One of the most difficult things for the participants during the process with Dignitas is the need to receive the organization’s approval for their request to receive aid-in-dying, mainly based on medical criteria. One participant felt the need to “physically impress” the organization, and as such decided to have dental implantations and take care of her hair prior to travelling to Switzerland. Some participants criticized the fact that only terminal conditions such as cancer, or chronic diseases with no cure such as Parkinson's disease or multiple sclerosis, are approved. Another participant expressed her concern that while her medical condition is terminal for her, “it may not be *for them*.” For D., who felt like she had been living twenty years of hell, this is unfair. In her interview, she expressed why she deserves their help more than terminal patients: "Nobody is entitled to say to another person, ‘No. Not you. You should continue fighting.’ If a person wants to die… and is sure… then let him die."

Another distinction that emerged from the interviews relates to mental disabilities versus physical ailments. It seems that it is easier to receive approval from Dignitas when referring to physical symptoms rather than mental suffering, perhaps as the latter is more subjective and may raise concerns regarding the applicant’s ability to understand the implications of such a request. This was expressed by participants who had insufficient evidence of their physical suffering to add to their mental issues.

In addition to "fitting" the "right" criterion, participants referred to encountering great difficulty in obtaining medical records from their doctors for this purpose. One participant explained how when she approached her doctor—who had even hinted at such an option in the past, refused to write a letter to Dignitas, stating that as the illness was not terminal, she cannot help her. Another participant shared her struggle in finding a doctor who would agree to support her request:

I was constantly looking for a doctor, but it was like looking for a needle in a haystack. I thought of going to doctors in Tel Aviv who are more open minded… So, should I go to see a doctor privately?... Could you please write me a letter? If he doesn’t know me, how will he be able to... It would take hours and what doctor would sit with me for hours? Even a private medical consultation only lasts half an hour or an hour… I have an idea how to lie to a doctor…

In this study, participants vary in the extent to which they *shared information about their plan* with others. One participant admitted that sharing her plan to go to Switzerland with others could result in attempts to dissuade her. On the other hand, she said telling people is important so that they will not be surprised. Another participant mentioned she had not shared her plan with anyone, because this would be very upsetting for those who are close to her (her brother or son). A third participant wanted to keep our talk secret so that her caregiver would not find out about her plan, as "she is Christian and a very, very simple woman".

Most of the participants who shared their plan with other said that they had received positive feedback. As conveyed by C.:

It works, the way I tell them, people get it. They don't refer me to mental care and don't suggest drugs, antidepressants. They accept what I’m telling them. They may say to themselves, ‘OK, she doesn't really mean this,’ but I try to say it again and again… If I die tomorrow, people will say, ‘She always said that she wanted to die young or at some point.’

On the other hand, one participant who shared her plan with her close friends was met by fear or lack of support, which made her stop sharing. She heard comments like ‘she’s depressed’ or ‘she could still be recovering’ (from cancer). A fourth participant concealed his plan from his wife, because, in his opinion, "She can't help me with this, she won't agree. She’ll never agree to me ending my life."

Some participants referred to their family members as a source of either positive or negative input. E, for example, shared that although her son actually lives in Switzerland, she does not want to tell him about her plan or have him there when she arrives in the country: "He won't accept this… I know what his reaction will be. It will very unpleasant and aggressive. I don’t even know how to update him *post factum*. Maybe I will write him [a letter]." H., on the other hand, had already shared her plan with her only son, who at first did not want to be a part of this. B.’s family "understood" her decision without trying to dissuade her. G. had received a complex response:

My sister accepts this and so does her daughter, especially as she’s already 40 years old. It’s interesting as my niece is a married woman with two grown children and her husband's brother hung himself. Because of this, it was harder for my niece to hear about my plan… Her husband even said to me: ‘As long as my children are alive, you cannot do this. When they get married you can do this.’ He is not encouraging me to do this, but I was always independent and did whatever I wanted.

The most aggressive response was by one of I.’s sons when he found out that his mother had registered with Dignitas:

At one point my son had to take me in his car and suddenly, in the midst of horrible anger, he started screaming at me and said he knows what I’ve done and why wasn’t I thinking about him.... He wouldn’t even really talk about it, so I don’t know his exact reasons. We didn't talk about this again after that.

I. reported that a different son of hers, on the other hand, reacted much less aggressively. When she received the envelope from Dignitas with a request for payment, this son told her to throw it away and she did. She added,

He was on my side all the time, but we didn't talk much about what everyone was feeling at that moment. Most of the time he cried when I told him I wanted him to be in touch with this firm (Dignitas). I was very sad, heartbroken.

It follows that seven major themes were obtained, including [1] how participants understand, find meaning, and position themselves in relation to the phenomenon of suicide tourism and their plan to travel to Switzerland to receive aid-in-dying: [2] health and functioning status; [3] feelings pertaining to survivorship and existence; [4] experience with the health sector; [5] attitudes regarding death and dying; [6] suicide; choosing death; and [7] choosing suicide tourism.

## Discussion

This study provides a microanalysis of the suicide tourism phenomenon, depicting the complex dynamics between and within countries and citizens regarding matters of life and death, and of biopower and thanatopower embedded in the more general regulations and legalization of aid-in-dying [43]. It constitutes new knowledge in that the themes identified by it are not only themes which are common in people seeking aid-in-dying, but refer to a unique phenomenon characterized by special conditions by which such a practice takes place. This phenomenon is enhanced by a personal and intimate wish to seek death without fear [44] for themselves or for their loved ones, who help them fulfill their goals following the broader social movement of "requested death" [45].

For some interviewees, speaking openly and honestly about these issues was not possible prior to the research. As such, the interviews offered a channel for the participants to air their views about aid-in-dying and suicide tourism, while also providing a means for gaining insights and new information regarding such plans that are usually inaccessible or unavailable to the public. In addition, the concept of keeping such plans from family members seen in this study (to prevent their distress or intervention and to avoid criminal charges) is in line with the findings of a similar previous study [29].

Based on these findings, it can be inferred that the large majority of research participants are strong, autonomous people who are not yet situated within relationships of dependency on family members or professional caregivers. They do not necessarily want to have their loved ones beside them when they die, and they see no significant meaning in perhaps dying in a foreign country to which they have no emotional or civil attachment. For most, traveling to Switzerland for aid-in-dying is part of a continuum for living freely and independently deciding about private matters that belong only and exclusively to themselves. Although for some, raising public awareness of the subject was a significant outcome of their plan, it was mostly perceived as a personal decision, not necessarily a political statement.

However, the participant's personal traits and desires are in sharp contrast to the official requirement to obtain approval for Dignitas to control their dying process or alternatively, to its tragic denial. This supports the paradox which has been explored in the literature, between the perception of aid-in-dying as a mechanism for fulfilling an individually-controlled death and its bureaucratic and materialistic characteristics that cultivate dependency and collaboration [46].

Numerous positive metaphors were raised by the participants to depict what aid-in-dying through this process means for them. Specifically, participants viewed their plan to travel to Switzerland as ensuring a non-violent, non-painful, and secure way to die, after considering and rejecting more unpleasant and unsafe alternatives for ending their lives. As in a similar study [29], participants in this study regarded aid-in-dying in Switzerland as a good death, because of its guaranteed outcome. Some compared this method of dying to suicide, the latter being perceived as negative, violent, and harmful to others. This is in line with the more general literature which suggests that contrary to suicide, which represents a "bad", uncontrolled, and sudden death, aid-in-dying symbolizes a pre-arranged, and caring death [47].

The way a person dies, including through suicide, can be defined as good, honorable, or aesthetic. Historically, choosing suicide indicated a person's social status and moral character. Aesthetic death was associated with courage and being resolute, contrary to a rash or impulsive act through popular means such as hanging [48]. The very detailed and strict Dignitas program and its comprehensive preplanning, which at times was criticized by the participants, requires the participants to double check their desire to end their lives, thereby ensuring that their request is not impulsive but rather encompasses much consideration.

The "beautiful death" depicted in the alternative offered by the travel to Switzerland is also contrasted with a state of "disadvantaged dying" [48, 49], characterized by imposing a burden on the participants' loved ones, especially with a prolonged dying process. The participants’ fear of being a burden or causing distress to family members, which goes hand in hand with their being strong independent individuals, is in line with how people imagine a desirable death [19].

Similar to other studies [19, 50], a strong focus on controlling death and dying, as well as their timing and how these are perceived by others, is also reflected in the study findings. The idea of control is addressed early on, with participants considering the many details required for this process, and through their sharing their plan, consulting, and seeking helps from others.

As with previous studies on aid-in-dying, this study found that suffering is understood within the subjective dimension, namely only by the person who is actually subjected to the disease, ailments, and disability [16, 23, 48]. Such conceptualization accords with the more general understanding of the notion of dying, whereby dying only occurs when the person herself is aware and accepting that death is an imminent event [50].

Moreover, participants in this study reflect the idea that pain and suffering are experiences from which one should escape before they require too much from them and before they diminish all signs of human dignity and sense of living. This concept is in line with people's wish to die in their sleep, not being aware of the actual dying phase [19]. However, one may argue that it is this state of affairs that exposes human vulnerability and calls for a giving and caring relationship that is reciprocal and central to a culture [2]. If one accepts this argument, then the findings presented in this study may reflect the fact that elimination or denial of suffering challenges the very essence of what it means to be human, to be a member of a community. Yet participants in this research did not convey such a complex relationship with their existentiality, regarding it instead as one-dimensional whereby the sole remedy for their situation is ending their own life and as soon as possible.

Most participants in this study referred to chronic illnesses, functional disability, and social isolation—all of which serve as common drivers of suicide [48]. As seen in previous studies, social isolation and loneliness were found to play a role in their decision to receive aid-in-dying [29, 51]. However, despite efforts to distinguish between suicide tourism and suicide, arguing that people who seek aid-in dying are not suicidal [5], a significant portion of the participants had experienced suicidal thoughts and had even previously attempted suicide, some more than once. They claimed that their decision to end their lives was a rational one, based on their sense of life quality and worth (or lack thereof). They criticized the alleged hierarchy between the various "reasons" for seeking aid-in-dying, the circumstances of the person at stake, the different ethical justifications: terminal illness versus other forms of illness; physical disabilities versus mental illness; existential versus physical pain and suffering; older candidates versus younger ones.

In this study, many of the participants criticized the criterion whereby only terminally-ill people can receive aid-in-dying—permitting only those suffering from unbearable and incurable medical circumstances to exercise their autonomy regarding how they die. As Ost and Mullock [52] argue, this view overlooks people's existential suffering where the individual feels 'tired', 'weary of life', or 'done with life', thereby violating the values of autonomy and compassion. Such situations render people in incapacitating states of despair resulting from the inner realization that life is futile and meaningless [53].

Paradoxically, in the case of suicide tourism, it is not only the physicians from the patient’s country of origin who must support the specific request for aid-in-dying (i.e., who must determine that the specific "type" of suffering and medical condition qualifies for such a request); Dignitas and the Swiss doctors must also reach the same conclusion [54]. Moreover, the so-called aesthetic, non-medicalized model of aid-in-dying in Switzerland (where aid-in-dying can be provided by lay people), is disguised by the unattractiveness and dominant medical control that acts in the background and is guided by medical criteria.

Contrary to a previous study [29], participants in this study did not perceive chosen method of death as sinful, nor was there a correlation with their religious beliefs. In fact, very few referred to religious concepts such as sanctity of life, belief, and God. Some were even critical of the religious impact on the legality of euthanasia and aid-in-dying, regarding such influence as a substantial interference in the person’s life. They confirmed previous findings whereby in Israel, religion plays an important role in the end-of-life debate [55], at times an even greater role than the law itself [56].

Content analysis of medical reports that were used as part of requests for aid-in-dying in Switzerland revealed a significant variance in the referring physician's awareness and support of this act. In turn, this reflects the challenge in avoiding legal prosecution in the country of original [57]. This study also found that some healthcare providers who were approached by the participants refused to provide support for the participants' request, due to legal risk or aversion from the proposed act itself, thereby imposing much difficulty on the participants. In some countries, the physicians' negative response to suicide tourism and lack of cooperation is backed by professional organizations, such as the British Medical Association (BMA) who states that "although to date there has been no prosecution for people accompanying others abroad to end their lives, doctors need to be aware of the possibility of legal and professional sanctions if they were to do so." [58] (p. 3).

## Conclusions

Individuals contemplating travelling for aid-in-dying show a strong desire to promote their plans, some even worry that they might be too late as they are becoming too physically impaired to carry out this act. The desirable approval or tragic refusal by Dignitas to participants' requests for suicide tourism enhances the paradox between the perception of aid-in-dying as a mechanism for fulfilling controlled death and its bureaucratic and materialistic characteristics specifically reflected in a paid, formalized approach to aid-in-dying that cultivate dependency and collaboration.

Future studies could therefore benefit from re-examining the conditions, resources, and challenges that such a unique population faces in advancing their autonomous wish to die, while struggling with their medical condition, poor quality of life, and even loss of hope.

## Limitations

Although the research design provides valid and reliable information, the study may have some limitations. First, the interviews and data analysis were conducted solely by the researcher. To overcome this limitation, the data were analyzed through a multi-layer approach, with the author ensuring reflexivity during the analysis. This was secured by taking personal notes throughout the study and interactions with participants that facilitated the continuous and systematic examination of subjectivity. Challenging issues that arose during the interviews were discussed and examined with my peers, especially members of the the Research Ethics Board. Moreover, given the sensitive topic and the rapport that was built with the participants (which in itself contributed to the development of the interviews), it is likely that the outcome provided in-depth meaning and rich context regarding the participants' experiences and attitudes [59]. Second, the study consists a relatively small number of participants. However, with qualitative research, the sample size is determined by the context. In this study, this is attributable to the research population, i.e., people who are planning to travel to Switzerland to end their lives, and as such are not easily accessible or identifiable due to legal threats, possible interventions by their loved ones, or significantly decreased health that prevents them from participating in the research. Yet regardless of the small sample, the participants' stories, experiences, and attitudes provided a real depth of understanding, rather than a mere breadth of the suicide tourism phenomenon, as perceived by those who are actually engaged in it. This therefore provides a highly instructive understanding of the phenomenon and the sources that drive it [60].

## Data Availability

The datasets generated and/or analysed during the current study are not publicly available since these are private data obtained through in-depth interviews but are available from the corresponding author anonymously on reasonable request.
